# High efficiency integration of three-dimensional functional microdevices inside a microfluidic chip by using femtosecond laser multifoci parallel microfabrication

**DOI:** 10.1038/srep19989

**Published:** 2016-01-28

**Authors:** Bing Xu, Wen-Qiang Du, Jia-Wen Li, Yan-Lei Hu, Liang Yang, Chen-Chu Zhang, Guo-Qiang Li, Zhao-Xin Lao, Jin-Cheng Ni, Jia-Ru Chu, Dong Wu, Su-Ling Liu, Koji Sugioka

**Affiliations:** 1 CAS Key Laboratory of Mechanical Behavior and Design of Materials, Department of Precision Machinery and Precision Instrumentation, University of Science and Technology of China, Hefei 230026, China; 2 School of Life Science, University of Science and Technology of China, Hefei, Anhui, 230027, China; 3 Laser Technology Laboratory, RIKEN, 2-1 Hirosawa, Wako, Saitama 351-0198, Japan

## Abstract

High efficiency fabrication and integration of three-dimension (3D) functional devices in Lab-on-a-chip systems are crucial for microfluidic applications. Here, a spatial light modulator (SLM)-based multifoci parallel femtosecond laser scanning technology was proposed to integrate microstructures inside a given ‘Y’ shape microchannel. The key novelty of our approach lies on rapidly integrating 3D microdevices inside a microchip for the first time, which significantly reduces the fabrication time. The high quality integration of various 2D-3D microstructures was ensured by quantitatively optimizing the experimental conditions including prebaking time, laser power and developing time. To verify the designable and versatile capability of this method for integrating functional 3D microdevices in microchannel, a series of microfilters with adjustable pore sizes from 12.2 μm to 6.7 μm were fabricated to demonstrate selective filtering of the polystyrene (PS) particles and cancer cells with different sizes. The filter can be cleaned by reversing the flow and reused for many times. This technology will advance the fabrication technique of 3D integrated microfluidic and optofluidic chips.

Microfluidic chips are systems in which microfluidic channels are integrated with microcomponents possessing some functionalities including pumping^1^, mixing^2^, sorting^3^, trapping^4^, detection, and sensing, thus have attracted great attentions due to their broad applications in chemistry, biology, medicine, and pharmaceutics. They revolutionized chemical and biological researches in the 21 century^5^,^6^,^7^, since they offer distinct advantages of ultra-low reagent consumption, low cost, high-speed, high integrity, portability, and miniaturization^8^,^9^. These outstanding advantages have greatly promoted the rapid development of this technology to integrate functional devices into microfluidic chips towards high functionalities^10^. For example, ultraviolet(UV)^11^, e-beam^12^, X-ray lithography^13^, and nanoimprinting^14^ have been successfully applied for the fabrication of multifunctional microfluidic chips. In principle, these methods are available only for creation of planar microfluidic devices and face difficulty for integrating functional 3-dimensional (3D) complex microstructures inside a given microfluidic chip. In addition, the nonplanar microchannel networks also limit their extensive applications to the integrated chip fabrication. To realize 3D multifunctional microfluidic chips, a variety of novel methods including direct-write assembly of a fugitive organic ink^15^, combining holographic lithography and photolithography^16^, and using soft paper/polymer composite by simple bending and stretching^17^ have been developed. However, it is still difficult to integrate complex 3D microstructure in a designable, flexible, and controllable way. Therefore, it is highly desirable to develop a new processing technology to fabricate and integrate the functional 3D microchips.

Femtosecond laser microfabrication^18^ by two-photon polymerization (TPP)^19^,^20^,^21^,^22^ is a promising method to reach this end due to its distinct advantages such as the programmable designability, 3D processing capability, high spatial resolution, and the diversity of usable materials. TPP has been used to fabricate high numerical aperture microlens arrays^23^, high efficiency zone plates^24^, microbulls^25^ and micro-chain structures^26^ on surface. In addition, TPP can also integrate a variety of 3D microstructures such as a overpass^27^, micromixer^28^, microfilter^29^ and center-pass optofluidic microlens array^30^, into a microfluidic channel for guiding different fluids, high efficiency mixing of different fluids, controllable filtering of particles and cell counting, respectively. Although TPP has been regarded as a powerful method for functional integration of microfluidic chips, from the viewpoint of practical applications, the processing time will be the most significant obstacle of TPP due to its single-point writing scheme. Several methods for shortening the processing time of 2PP have been developed such as surface-profile scanning^26^ and multifoci scanning^31^,^32^. The surface-profile scanning followed by additional UV irradiation has been proposed to reduce the processing time for formation of structures with large interior volumes, while it’s not effective for high porosity or thin structures^26^. Parallel multi-beams produced by microlens array^31^ and a diffractive beam splitter^32^ can also increase the fabrication efficiency, but this method can’t precisely control the position of every-focus for arbitrarily arrangement of multi-foci. Holographic femtosecond laser direct-writing by switching hologram data on the spatial light modulator (SLM) can overcome these limitations. In 2011, Chichkov *et al*. used this technology to create 16 micro-Venus structures by TPP multifoci with a SLM which significantly reduced the processing time by a factor of 1/16^33^. In 2013, our group advanced the technology for parallel fabrication of aspheric microlens arrays with excellent optical performance^34^. These have proved that multifoci 2PP scanning with the SLM will solve the time-consuming issue in 2PP integration for lab-on-a-chip fabrication, although it has never been used for 3D integration of microfluidic chips due to the complex microenvironment in the deep or curved microchannels.

In this work, we utilize femtosecond laser multifoci 2PP to integrate 2D and 3D microstructures in a given ‘Y’ shape channel with holograms displaying on a SLM for the first time. In order to integrate high quality microstructures into a microfluidic chip, the laser powers, the prebaking times and the developing times in the channel and on the surface were compared to optimize the process conditions. Then, a variety of 2D-3D microstructure arrays with high surface quality, e.g., 2D ‘LOC’ characters, ‘Cross’ and ‘Svastika’ patterns, and 3D layered structures, hierarchical gecko palm-like pillars and standing helixes have been formed both on the surface and in the channel. The total scanning time for creating 3 × 3 arrays of 3D layered structure is 11 min, which is much shorter than that (99 min) by a single beam. Then, functional micro-filters are designed and integrated into the channel by five- or seven-foci scanning with an SLM. The hole size of the filters can be precisely controlled by the laser power. The filter is tested with a suspension with diameters of 2.8 μm, 5.2 μm and 13.0 μm polystyrene spheres in alcohol solution and can completely filter 13.0 μm beads out. Finally, the filter demonstrate the capability of separating the cancer cells.

## Results and Discussion

### Multifoci laser fabrication of 2D and 3D microstructures

**Figure 1:**
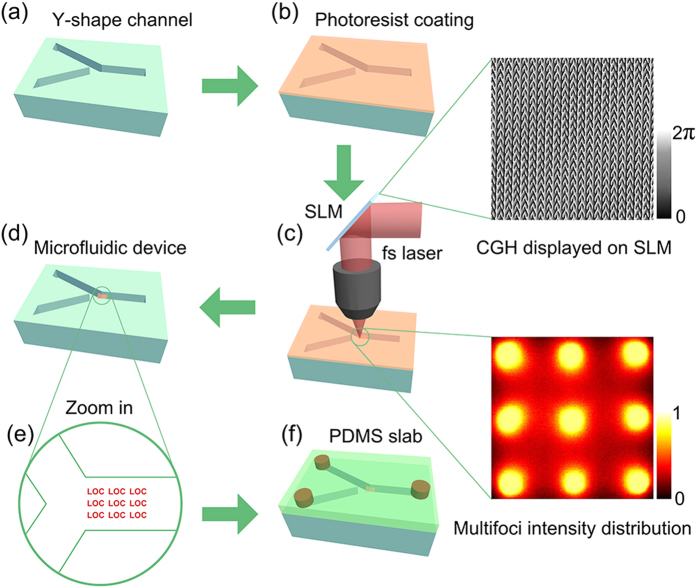
Schematic illustration of fs laser multifoci parallel integration of microstructures inside a ‘Y’ shape microchannel. (**a**) A ‘Y’ shape microchannel fabricated in photosensitive Foturan glass by femtosecond laser-assisted wet etching. (**b**) Surface coating with photoresist SZ2080. (**c**) Fs laser mulfoci (9 foci) parallel integration of microstructures inside the given microchannel. The enlarged images are a CGH displaying on the SLM for generating 9 foci pattern and a 3 × 3 foci intensity distribution respectively. (**d**) Developing in 1-propanol to wash away the unpolymerized resin. (**e**) Magnified schematic image of the integrated microstructures (e.g., 3 × 3 array ‘LOC’) inside the microchannel. (**f**) Sealing the microchannel with a PDMS slab to form a microfluidic chip.

**Figure 2:**
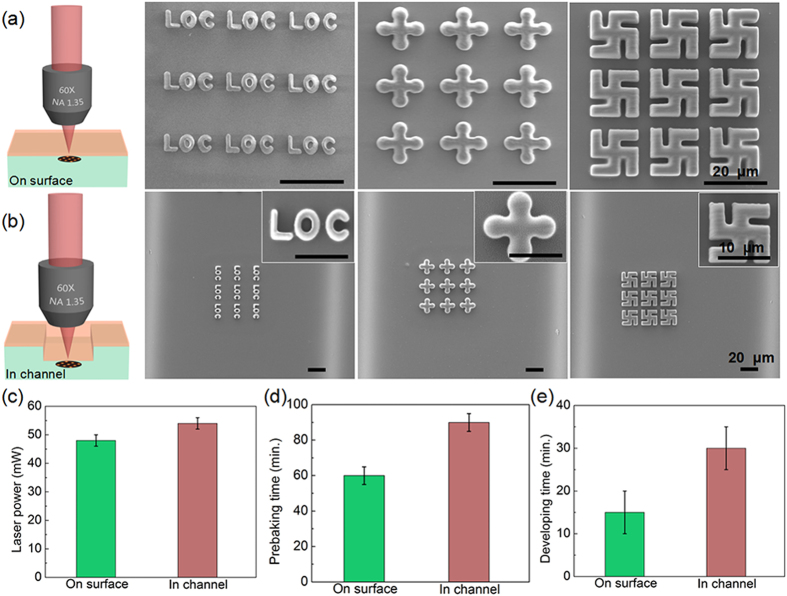
Quantitative investigation of three crucial parameters for high efficiency fs laser multifoci 2PP and high precision 2D microstructures on surface and in channel, respectively. (**a,b**) Schematic illustrations of high efficiency fs multifoci processing and the fabrication results of 3 × 3 arrays of 2D ‘LOC’ characters, ‘cross’ and ‘Svastika’ patterns on surface (**a**) and in channel (**b**). (**c–e**) Systemic comparison of optimal laser powers (**c**), prebaking times (**d**) and developing times (**e**) for 9 foci parallel fabrication on surface and in channel.

**Figure 3:**
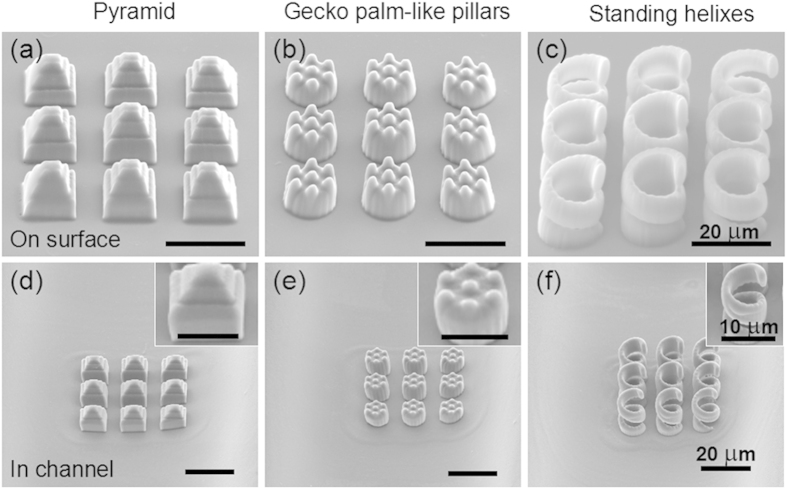
A series of 3D microstructures fabricated by high efficiency SLM-based fs laser microfabrication on surface and in channel. (**a–c**) shows 3 × 3 arrays of pyramid, hierarchical gecko palm-like pillars and standing helixes on surface, respectively. (**d–f**) shows the same microstructures fabricated in channel.

As a proof-of-concept demonstration, a 3 × 3 array with a spot interval of 20 pixels was utilized to integrate microstructures into the channel in parallel. Figure 1 is a schematic illustration of fs laser multifoci parallel integration of microstructures inside a ‘Y’ shape microchannel. A ‘Y’ shape microchannel was first fabricated in photosensitive Foturan glass by femtosecond laser-assisted wet etching (Fig. 1a and Supplementary Fig. S1). Then the glass surface coated with photoresist SZ2080 (Fig. 1b). Next Fs laser mulfoci (9 foci) parallel fabricated microstructures inside the microchannel (Fig. 1d). Developing in 1-propanol to wash away the unpolymerized resin, the microstructures remained in the channel (Fig. 1c–e). The microchannel was sealed with a PDMS slab to form a microfluidic chip (Fig. 1f) and alcohol with purple food dye as a media fluid was filled into the channel to verify that the microfluidic chip was sealed completely (Supplementary Fig. 9S and video 1). Figure 1c show a computer generated hologram (CGH) for producing 9 foci and a resulted 3 × 3 foci intensity distribution captured by a CCD camera, respectively. In order to fabricate high-quality polymer microstructures into the microchannels, the processing conditions for multifoci 2PP need to be optimized. The conditions for TPP in a microchannel will be different from those on a flat surface. Specifically, Wang *et al*. pointed out that the working distance of the 60X oil lens with NA of 1.35 was very short, so that the excess resin had to be moved away by straight-edged cover slide to protect the lens and higher laser power was required in channel than on the flat surface because of the possible ‘wall effect’^35^. Osellame *et al*. developed a optimized procedure including a preliminary prebaking of the SZ2080 resin for 3 mins at 80 °C, then prebaking the chip with resin at 105 °C for 90 mins^29^. In our experiment, we found there are three main factors which will influence the fabrication: laser power, prebaking time and developing time. Firstly, the optimum laser powers on surface and in channel were systematically investigated (Fig. 2c). To fabricate the same structures, the needed laser power of 9 foci is about 54 mW in the channel while about 48 mW, on the surface. Generally because of the ‘wall effect’ and thicker resin in channel, the laser power needed in the channel is 1.1 ± 0.5 times larger than that on the surface, which is consistent with the result by Wang^35^. The prebaking time and the developing time were also investigated. The prebaking time was about 90 min in the channel while it was 60 min on the surface. This is because that the resist in glass microchannel is so thick that heat transfer is slow and a longer prebaking time is needed to ensure that the whole solvent can be evaporated from the resist. Similarly, the developing time on surface was only 15 min. In contrast, a longer time of 30 min was needed for the in-channel to ensure the completion of the developing process. In addition, there are three key experimental factors (Supplementary S4, S5, S6 and S7) hindering the multi-point parallel integration in channel which are described in detail in the supplementary information. After optimizing the fabrication parameters, a variety of high quality micropatterns, e.g., a 2D pattern arrays of ‘LOC’ characters, ‘cross’ and ‘Svastika’ patterns were realized both on the surface and in the channel (Fig. 2a,b). We can see that the quality of the structures in the channel is comparable to that on the surface. In addition to 2D structures, arrays of 3D layered structures, hierarchical gecko palm-like pillars and standing helixes were also fabricated in the ‘Y’ shape channel and on the surface (Fig. 3a,b). The fabrication time of the arrays of layered structures is only 11 min using multifoci while it reaches as long as 99 min using single-focus. The above structures can be applied for biomimetic superhydrophobic surfaces, dry adhesion^36^, and refractive index sensing, respectively. These results demonstrated that this SLM-based parallel method was effective to integrate various functional microdevices inside a microfluidic chip with high speed and accuracy.

### High-efficiency multifoci integration of 3D functional filters

**Figure 4:**
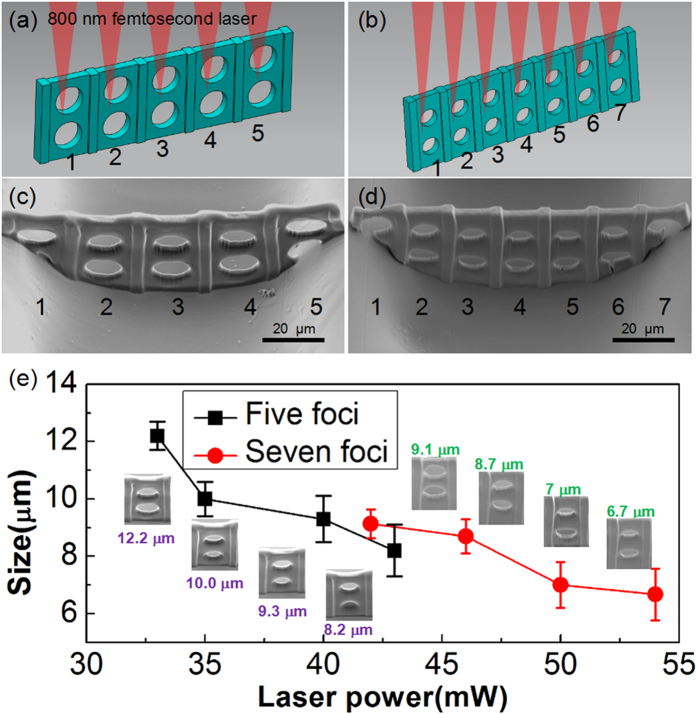
Multifoci integration of different microfilters inside the microchannel and dependence of the hole size formed in the filters on the laser power. (**a,b**) Schematic illustrations of five foci (**a**) and seven foci (**b**) integration of microfilter. (**c**,**d**) show the SEM images of the microfilters integrated in the microchannel by using five foci and seven foci, respectively. (**e**) Dependence of the hole size formed in the filter on the laser power. The larger the laser power, the smaller the hole size. The insets show the corresponding SEM images.

Based on systematic investigation on the optimal experimental parameters performed above, we attempted to integrate functional microdevices into the microfluidic chip. In particular, porous filter is one of the most important devices in a LOC system and has a variety of applications in the separation of micro/nanoparticles with different sizes, individual populations of cells from heterogeneous samples or circulating tumor cells (CTCs), and platelet separation from blood^37^,^38^,^39^. Here, we designed a typical structure 3D screen-like filters (Fig. 4a,b). From the SEM images in Fig. 4c,d, it is clearly seen that the bottom of the channel is not flat. Even the curvature is so big, various 2D-3D structures and functional microfilters can be fabricated with high quality, which verifies the powerful fabrication capability of the SLM-based parallel fabrication technique on various plat or curved surfaces. This is very beneficial for practical microfluidic integration in channels because the bottom shapes of channel prepared by the typical fabrication method is usually not absolutely flat. The time for fabricating the 13 μm pore filter with conventional single-focus 2PP scanning for a 10 ms exposure time at each spot is estimated to be 75 min. In contrast, the fabrication time is only 15 min with SLM-based 5 foci. Likewise, for the 10 μm-pore filter with 7 foci, the fabrication time is only 9 min while 63 min, with single-focus (Supplementary Table S1). Moreover, the pore size of filter can be precisely controlled by adjusting the laser power. For example, by changing the laser power of five foci from 33 mW to 43 mW, the diameter of the pores in the filter could be controlled from 12.2 μm to 8.2 μm (black line in Fig. 4e). As the power increases, the pore size decreases because of the increasing size of the voxels^40^. The size of the filter with seven pores can also be varied from 9.1 μm to 6.6 μm (red line in Fig. 4e) by controlling the laser power. These controllable sizes can be adopted for flexibly filtering micro-particles or bio-cells with different sizes.

### Characterizing the function of microfiltering by Polystyrene microbeads

**Figure 5:**
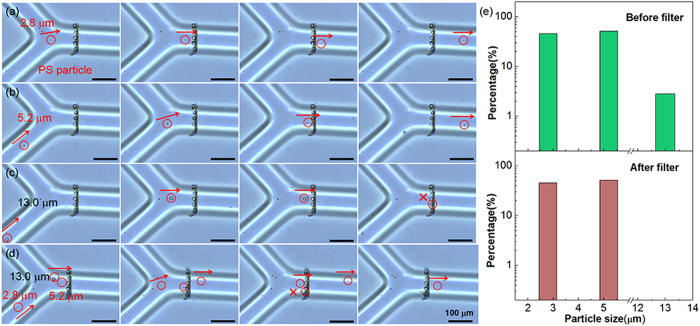
The filtering function (PS particles) of a 12.2 μm-pore microfilter. (**a,b**) A 5.2 μm and 2.8 μm PS particle passing through the microfilter easily. (**c**) A 13.0 μm PS particle was blocked by the filter. (**d**) The three different sizes of particles flowing to the filter. The smaller particles pass through the filter while the larger ones are blocked with 100%-success rate. (**f**) Numbers of the microparticles for different sizes of 2.8, 5.2, and 13.0 μm before and after the filter.

To verify the function, a microfilter with 12.2 μm pores (Fig. 4a,c) were fabricated with five-foci to filter beads with different sizes. Herein, polystyrene (PS) particles (a coefficient of variation of 3%) with diameters of 13.0 μm, 5.2 μm, and 2.8 μm (Huge Biotech Corp, Shanghai) were mixed in alcohol solution for evaluating the filtering performance. Figure 5a–c showed time-lapsed optical microscopy images of the filtering function of the above three different diameters of particles, respectively. It can be clearly identified that the particles smaller than the pore size can easily pass the filter. On the contrary, bigger ones are filtered out. When the three different sizes of particles were simultaneously introduced into the channel (Fig. 5d), the 13.0 μm particle is blocked by the filter while the 5.2 μm and 2.8 μm particles can freely pass through the filter. Statistic results (Fig. 5e) show that 100% of particles with size larger than 12.2 μm have been successfully filtered out. In contrast, particles smaller than 12.2 μm can completely pass through the filter, presenting an excellent filtering capability (Supplementary video 2). It is noticed that the PS particles blocked by the filter adhere to the filter or the microchannel walls (see Fig. 5). This will restrict the small particles to further passing through. This can be resolved by reversely flowing the alcohol to remove the blocked particles (Supplementary Fig. S10, Supplementary video 3). By injecting the alcohol through the filter from the outlet with a syringe, the particles have been removed from the filter and walls, while the filter still remains. This also proves the robustness of the microfilter, and then indicate that this integrated microfluidic chip can be reused for many times.

### Application of the microfilter for seperating cancer cells

**Figure 6:**
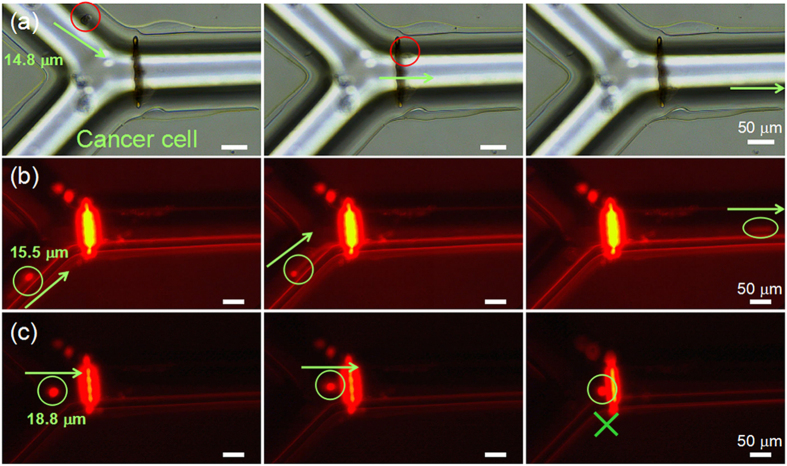
Filtering the cancer cells by the 12.2 μm pore microfilter. (**a**) Optical microscope images of 14.8 μm SUM 159 cancer cell passing through the filter. (**b**) Fluorescence microscope images of 15.5 μm SUM 159 cancer cell passing through the filter. The cell on the right of Fig. 6b looks like elliptical because of the rapid liquid flow in the channel. (**c**) Fluorescence microscope images of a 18.8 μm SUM 159 cancer cell flowing to the microfilter, being blocked and then adhering to the filter.

Circulating tumor cells (CTCs) in peripheral blood of cancer patients have important applications for metastatic detection and treatment monitoring^41^. The isolation of CTCs from blood cells can be utilized for cancer prognosis, assessment of tumor sensitivity to anticancer drugs, and anticancer therapy^42^. Here, we demonstrated that the filter can separate SUM 159 triple-negative breast cancer cells^43^. Figure 6a shows optical microscope images of a 14.8 μm SUM 159 cancer cell passing through the microfilter with 12.2 μm pores (Supplementary video 4). Because the cell can emit the fluorescence, fluorescence microscopy was used to clearly observe the filtering ability. As shown in Fig. 6b,c, the 18.8 μm cell was blocked while the 15.5 μm cell passed through the filter. The 14.8 and 15.5 μm cells passed through 12.2 μm filter due to the cell deformable ability. However, the 18.8 μm cell was blocked because it has a larger size of nuclei which were much more difficult to be deformed than the cytoplasm. The filter may find broader applications in separation of circulating tumor cells (CTCs) and blood cells.

## Conclusion

This work presents development of a simple, rapid, and effective tool for high-efficiency integration of 2D and 3D microstructures inside a given microchip by SLM-based multifoci parallel laser scanning technique. By using multifoci strategy, the fabrication time by 3 × 3 foci arrays can reduced by a factor of 1/9 than that by the conventional single point scanning. Optimized laser power, prebaking time and developing time were determined for high-quality fabrication in the channel. By adjusting the laser power, pore size formed in the microfilters were well controlled. The filters have demonstrated the ability in separating PS particles and breast cancer cells with different sizes. By reversing the flow direction, the filters can be cleaned and be reused for several times. The fs laser multifoci 2PP fabrication not only retains the advantages of 2PP such as 3D processing capability, programmable designability, high accuracy and spatial resolution, but also achieves a sufficient speed for practical use. As a proof-of-concept demonstration, a 3 × 3 foci array used in this work can increase the processing speed by a factor of 9. Here, the number of foci is limited by the channel width (~100 μm). The fabrication efficiency can be enhanced by factors of 100 and 10000 if more foci e.g., 10 × 10, 100 × 100 were used in a wider channel (>500 μm). In the future, more functional devices, such as microlens arrays, waveguides, photonic crystals and cell scaffolds will be rapidly integrated inside the microfluidic chips. In order to further reduce the fabrication time, we will try to design 3D light distribution to directly create 3D total microstructures in 1 exposure. We believe that this technology will offer new possibility for LOC device fabrication in terms of integration of more advanced functions in microfluidic chips in a facile and highly efficient manner, and will find wider applications in chemical and biological study.

## Methods

### Fabrication of ‘Y’ shape channel

The open microfluidic channel was fabricated by femtosecond laser-assisted wet etching^44^,^45^,^46^. Firstly, a commercial photosensitive Foturan glass (Schott Glass Corp.) was irradiated by a focused fs laser using an objective lens with a numerical aperture of 0.46. The laser-treated sample was then annealed in a furnace to form a crystalline phase of lithium metasilicate. The annealed microchip was followed by chemical etching in an ultrasonic bath with a 10% hydrofluoric acid (HF) solution to selectively remove the crystalline phase. At last, the sample was treated with a second annealing to improve the surface quality. The microchannel has a width of about 106 μm and a depth of 35 μm (see Fig. S1 in the ESI).

### Multifoci integration of microstructures

The clean glass microchip was firstly coated with a hybrid organic-inorganic sol-gel SZ2080 (IESL-FORTH,Greece)^29^ which has the advantage of high optical quality, good post processing inertness and mechanical stability, and low shrinkage properties on the glass surface. Next, the microchannel coated with SZ2080 resin was prebaked at 100 °C for 90 min to completely remove the solvent. In our experiment, the laser source was a mode-locked Ti:sapphire ultrafast oscillator (Coherent, ChameleonVision-S) with central wavelength at 800 nm, pulse duration of 75 fs, and repetition rate at 80 MHz. After the beam collimation and the power attenuation (Supplementary Fig. S2(a)), the laser beam was incident on a reflective liquid crystal SLM (Holoeye, Pluto NIR-2, resolution of 1920 × 1080, pixel pitch of 8 μm, and diagonal of 0.7 in.) on which designable computer generated hologram (CGH) with 256 gray levels could be displayed (Fig. 1d). Optimum CGHs with 1080 × 1080 pixels in the center of the SLM were designed using a weighted Gerchberg-Saxton (GS) algorithm for multifoci pattern generation^34^. The first-order of the diffracted beam in the phase-modulated laser was utilized to form an desired multi-spot pattern, and the zero-order beam at P plane^33^,^34^ was eliminated by vertically offsetting the multi-spot pattern in 120 pixels from the center (Supplementary Fig. S2(a) and (b)). Then the phase-modulated laser beam was focused by a 60X oil immersion objective lens (NA = 1.35). The glass sample was mounted on a three-axis piezoelectric motion stage (Physik Instrument, E545) with a nanometer resolution and a 200x200 ×200 μm moving range. After laser scanning, the sample was developed in 1-propanol for 30 min to wash away the unpolymerized resin (Fig. 1c). Finally, a cured PDMS (Dow Corning, US) was used to seal the glass microchannel (Supplementary video 1).

### Integration of microfilters

In order to rapidly integrate the filter into the microchannel, the filters were divided into 5 parts with 13 μm pores and 7 parts with 10 μm pores (Supplementary Fig. S8). Each part is solidified by a focal spot spitting by a pre-designed CGH (Supplementary Fig. S3) displaying on the SLM (Supplementary Fig. S3a,b). The width of the designed filter is a little wider than that of the channel to ensure that the filter blocks the entire cross section of microchannel. In addition, to enhance the filter robustness, thick rectangular columns (6 μm) are designed to be installed between each longitudinal array of pores.

### Preparation of polystyrene particles

PS particles (a coefficient of variation of 3%) with diameters of 13.0 μm, 5.2 μm, and 2.8 μm (Huge Biotech Corp, Shanghai) were mixed in alcohol solution for evaluating the filtering performance. We use alcohol to flow the particles by capillary force because of its good wettability to both the PDMS and glass. So we don’t need a syringe pump, at the same time, the flow speed can’t adjust precisely. The flow speed is about 400 μm/s in our experiment. The speed will greatly affect the performance of the filter. For example, if the speed is too slow (<200), the particles will adhere the glass surface. On the other hand, if the speed is too fast (>800), the particles will soon block the filter.

### Preparation of cancer cells

SUM159 triple-negative breast cancer cells obtained from Dr. Suling Liu have been extensively characterized^43^. The cell lines were grown in 60 mm petri dishes (Thermo Fisher Scientific, USA) using the recommended culture conditions as described previously^47^. Cells were treated with Trypsin-EDTA (Gibco, USA) solution after they became confluent for 1 minutes. Cell suspensions were then centrifuged at 1000 rounds per minute for 5 minutes in a centrifuge tube. New culture media were added after removing the supernatant and cells were resuspended by gently pipetting several times. Cell resuspension was diluted to proper density and added into the inlet of the channel. The cells are transfected with red fluorescent protein to help us observe the filtration.

## Additional Information

**How to cite this article**: Xu, B. *et al*. High efficiency integration of three-dimensional functional microdevices inside a microfluidic chip by using femtosecond laser multifoci parallel microfabrication. *Sci. Rep.* **6**, 19989; doi: 10.1038/srep19989 (2016).

## Supplementary Material

Supplementary Information

Supplementary Video 1

Supplementary Video 2

Supplementary Video 3

Supplementary Video 4
