# Supplementary material for: High efficiency integration of three-dimensional functional microdevices inside a microfluidic chip by using femtosecond laser multifoci parallel microfabrication
Source: Sci Rep. 2016 Jan 28;6:19989. doi: 10.1038/srep19989 (PMC4730193; doi:10.1038/srep19989)
Supplement: Supplementary Information [file srep19989-s1.pdf]

Supplementary Information for

High efficiency integration of three-dimensional functional microdevices inside a microfluidic chip by using femtosecond laser multifoci parallel microfabrication

Bing Xu[1], Wen-Qiang Du[1], Jia-Wen Li[1], Yan-Lei Hu*[1], Liang Yang[1], Cheng-Chu Zhang[1], Guo-Qiang Li[1], Zhao-Xin Lao[1], Jin-Cheng Ni[1], Jia-Ru Chu[1], Dong Wu*[1], Su-Ling Liu[2] and Koji Sugioka[3]

[1]Department of Precision Machinery and Precision Instrumentation, University of Science and Technology of China, Hefei 230026, China.

[2]School of Life Science, University of Science and Technology of China, Hefei, Anhui, 230027, China.

[3]Laser Technology Laboratory, RIKEN, 2-1 Hirosawa, Wako, Saitama 351-0198, Japan.

E-mail: huyl@ustc.edu.cn and dongwu@ustc.edu.cn

Table of Contents

- Figures S1 to S10 (pages 2 to 14)
- Tables S1 (pages 15)
- Captions for Supporting Videos 1 to 4 (page 16)

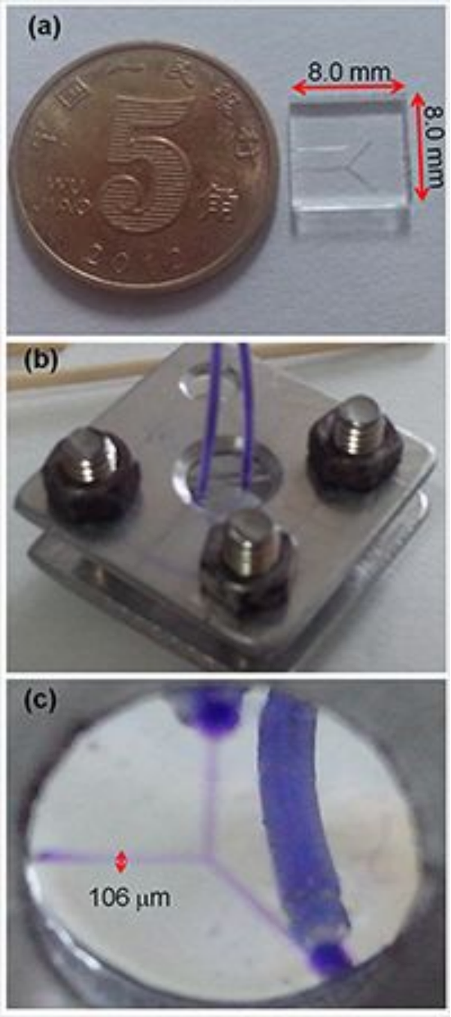

Figure S1. The 'Y' shape glass microchannel and the final microfluidic chip. (a) The glass is 8.0 mm length and 8.0 mm width. (b) shows the final device covered with a PDMS slab. (c) The magnified image of the 'Y' shape microchannel with 106 μm width and 38 μm height.

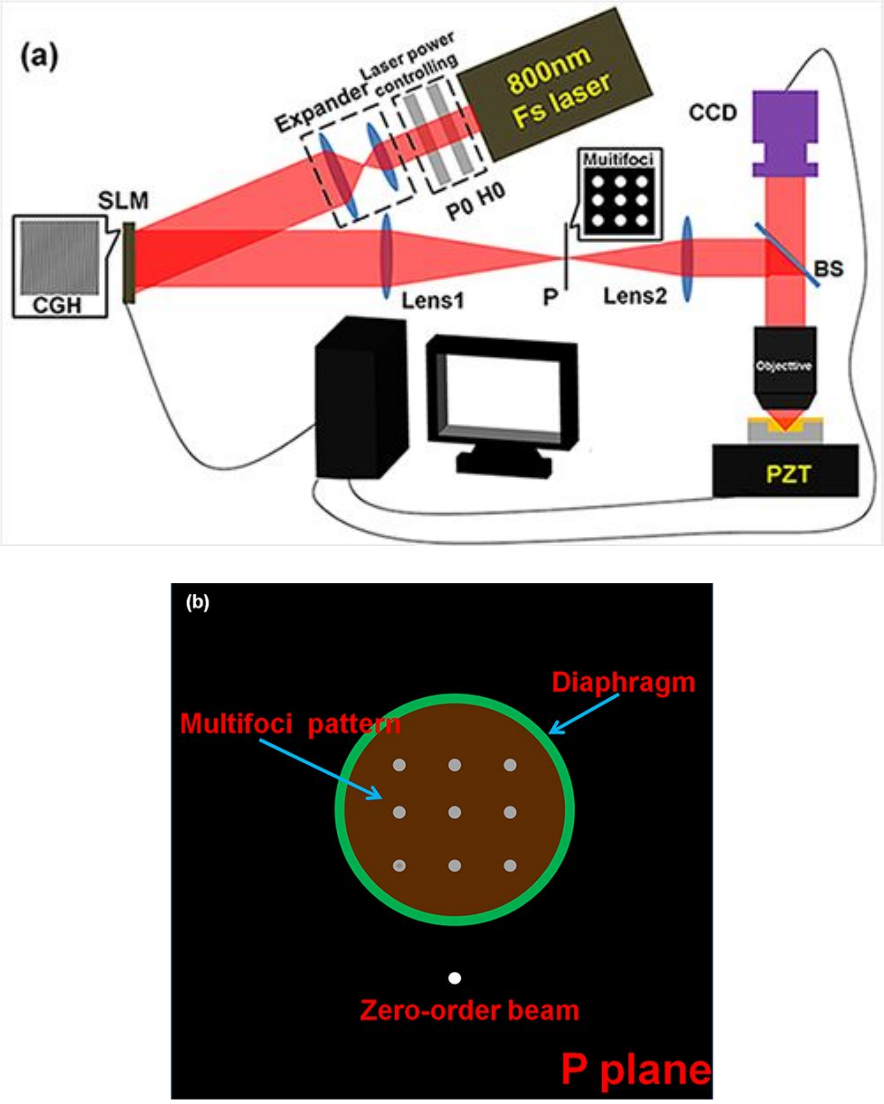

Figure S2. Schematic diagram of the laser system. (a) Schematic diagram of the laser system. H0 is a half wave plate and P0 is a Glan laser prim. (b) Magnification image of P plane. The zero-order beam with a higher light intensity than any ones of the multifoci (see from the picture) will strongly influence the fabrication results, so we vertically offset the multi-spots pattern 120 pixels from the center. In the 'P' plane, we add a diaphragm to block the zero-order beam, and the zero-order beam still remains in the diaphragm. It will not produce additional points or structures on the other regions of the sample. We defined the utilization efficiency by using the following formula:

$$\text{Utilization efficiency} = P_{multifoci} / P_P$$

Here, $P_{multifoci}$ and P_P represent the power of the desired multifoci and the power of the 'P' plane respectively. The utilization efficiency of 5 foci for filters fabrication is about 45%.

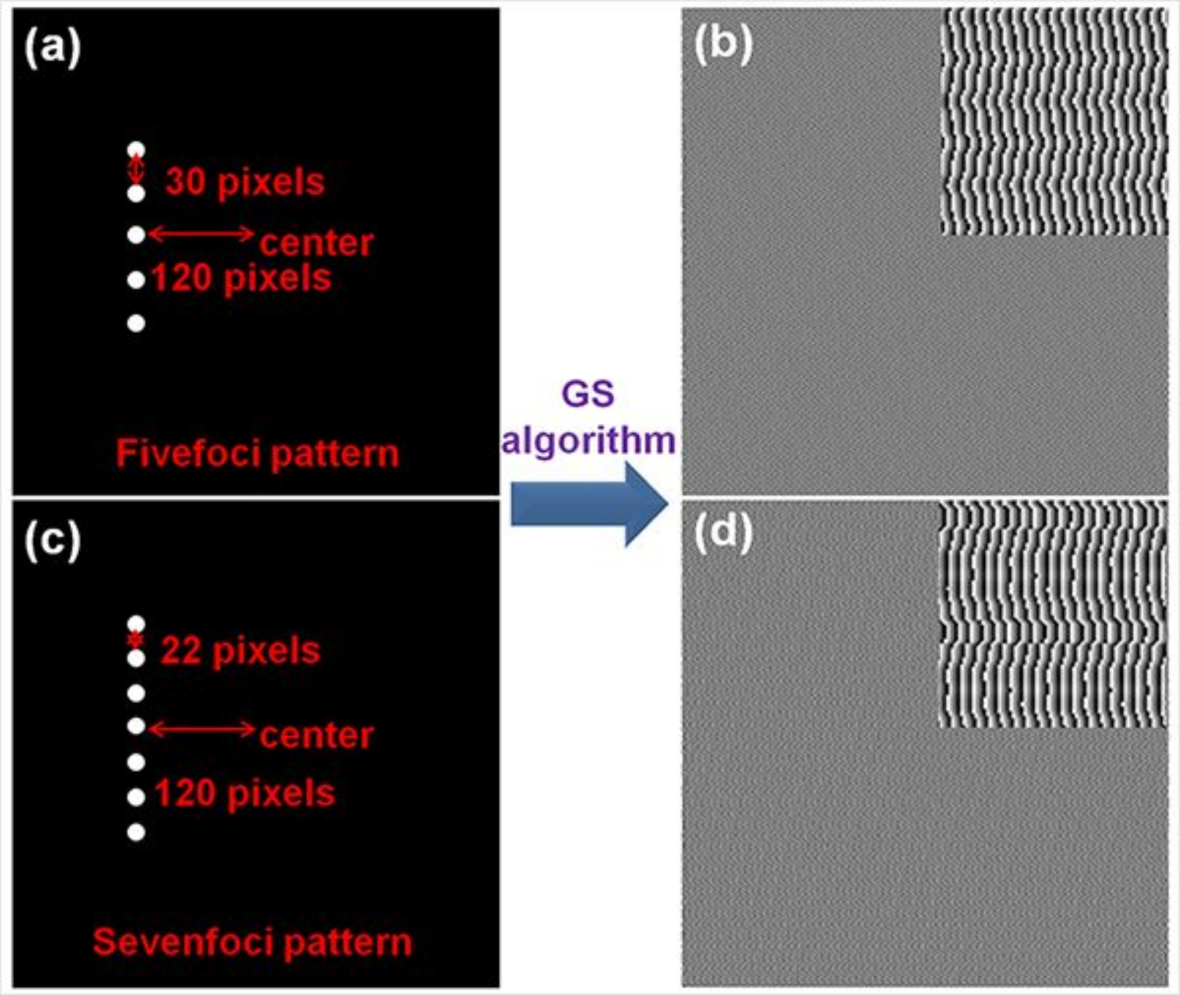

Figure S3. The generation of fivefoci and sevenfoci. (a) and (c) represented the five-spots (30 pixels interval) and seven-spots (22 pixels interval) pattern which are all vertically offsetting 120 pixels from the center. (b) and (d)) are the CGHs obtained by using a weighted Gerchberg-Saxton (GS) algorithm (an iterative 2-D Fourier transform calculation, 100 iterations) for five-foci and seven-foci generation, respectively. The multispot pattern were

firstly designed, then a weighted Gerchberg-Saxton (GS) algorithm was used to generate the desired CGH. The GS algorithm is reported before[1], and later a lot of researches have developed the method[2]. Here we use a weighted GS algorithm (100 iterations) reported by Hu[3] to get better uniformity. The intensity of each spot in desired multifoci pattern is monitored during iteration, and corresponding weighting factors are employed to update the original target field pattern in the next iterative loop.

1. Hayasaki, Y., Sugimoto, T., Takita, A. & Nishida, N. Variable holographic femtosecond laser processing by use of a spatial light modulator. Appl. Phys. Lett. **87**, 031101 (2005).

2. Gittard, S. D. et al. Fabrication of microscale medical devices by two-photon polymerization with multiple foci via a spatial light modulator. Biomedical optics express **2**, 3167-3178 (2011).

3. Hu, Y. et al. High-efficiency fabrication of aspheric microlens arrays by holographic femtosecond laser-induced photopolymerization. Appl. Phys. Lett. **103**, 141112 (2013).

There are **three key experimental factors** which hinder the multi-point parallel integration in channel.

(1) Firstly, in this experiments the flatness of the resin in the channel will greatly affect the fabrication structures. The resin coated on the glass usually was not very flat, just like undulating hills. This had weakened the foci uniformity a lot (Fig. 1a and b) while it will not happened in fabrication on surface. The fabricated filter had pores with different sizes (Fig. 1d). To solve this problem, the resin coated on the glass must be flat enough. In our experiment, we used a cover slip (a simple strategy) to remove off the redundant resin and ensured the flatness of the resin (Fig. 1c). From the SEM pictures (Fig. 1e), the flatness of the resin will greatly improve the quality of the filter.

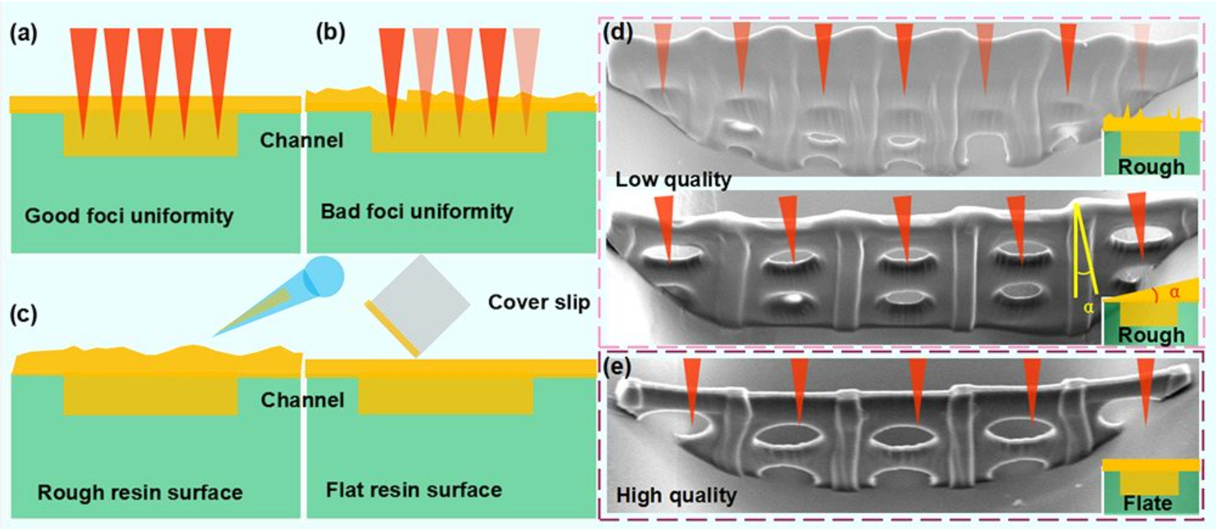

Figure S4. The influence of the flatness to the resulted structures. (a) and (b) shows good foci energy uniformity can be achieved by flat the resin. The brighter color represents the higher energy. (c) A cover slip is used to flat the resin and improve the quality of the resulted structures. (d) and (e) show the resulted structures with low quality and high quality by not using and using the flat resin respectively. The

inset is the schematic diagram of the resin surface. It is obvious that the flat resin can improve the quality of the filters.

(2) Secondly, the starting fabrication position is also a problem, especially in the channel. The resulted filter adhered the channel strongly if the laser foci scanning starting position was below the channel bottom (Figs. 2a and b right). Otherwise, if the starting position was above the channel bottom, the structures were very easy to detach from the channel and collapse (Fig. 2a and b left). In addition, if the position was much lower than the channel bottom (Fig. 2c left), the resulted filter had incomplete pores, and the incomplete parts were more than half of the pores (Fig. 2d left). In order to solve this problem, we use a simple strategy to align to the desired position on the chip by **observing the fluorescence intensity** (Fig. 3a, b and c) at the glass-resist interface. Fig. 5a and c show that the positions of foci are about 3 μm higher and lower than the glass-resist interface, respectively. If we choose the starting fabrication position just like Fig. 3, the resulted structures (Fig. 2b left) will detach. Also choosing the starting position just like Fig. 3c, the resulted filters will have incomplete pores (Fig. 2d left and middle). The position of foci in Fig. 3b is appropriate responding to the case of Fig. 2c right, and it can be seen that the fluorescence in Fig. 3a is strong, it weakens suddenly in Fig. 3b and it weakens further in Fig. 3c. The change of fluorescence intensity can be easily distinguished by a CCD camera (Fig. 3a, b and c).

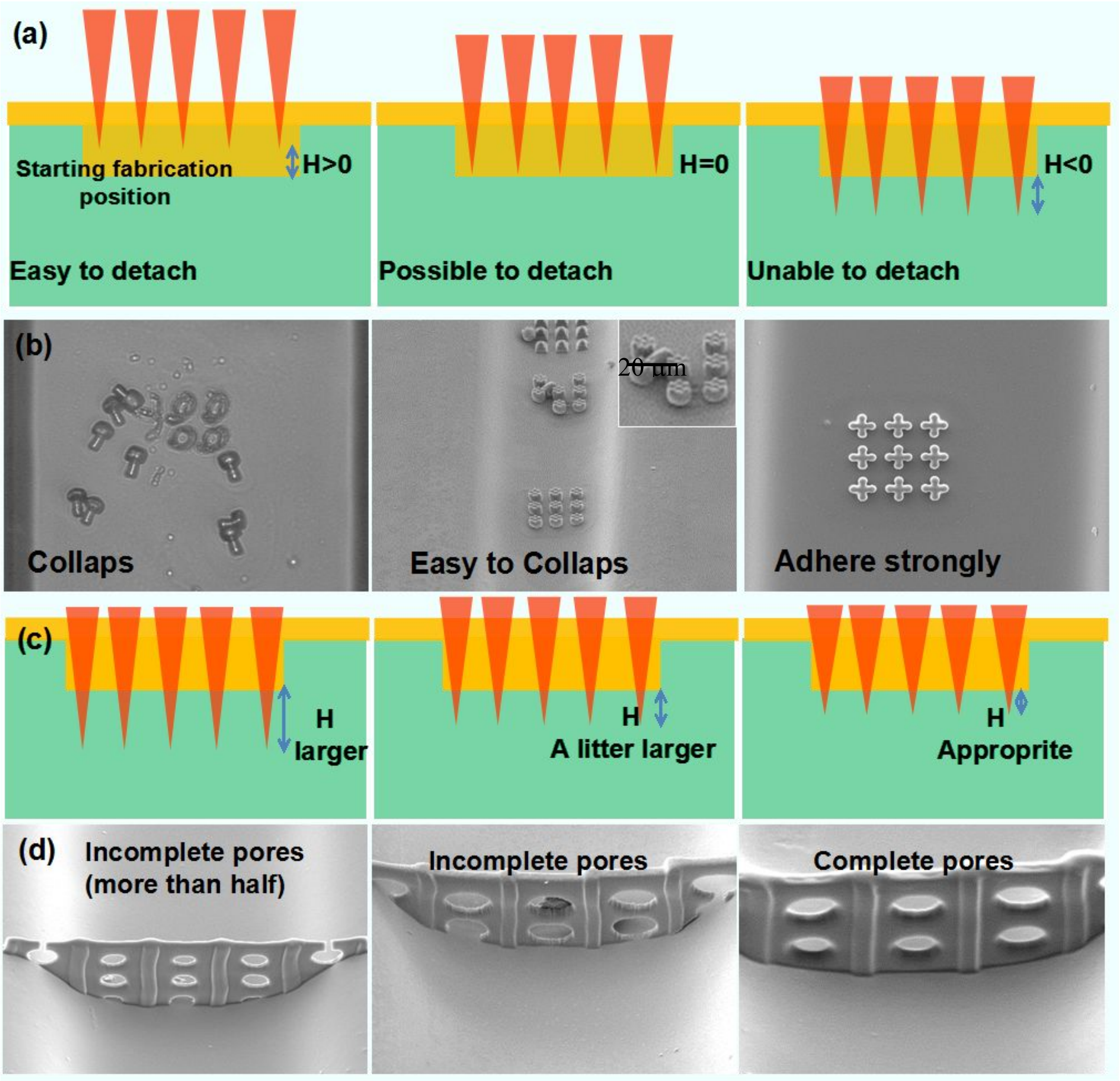

Figure S5. The right fabrication starting position. (a) shows schematic diagram of the starting position, it includes 3 conditions: higher than the channel bottom, just right on the channel bottom and lower than the channel bottom. And the resulted structures will collaps, collaps possibly and adhere strongly corresponding to the above 3 conditions showing in (b). (c) shows schematic diagram of the third condition which may have 3 subcases: lower, a little lower and a little bit lower apart from the channel bottom. (d) shows the resulted filter with incomplete pores (the missing parts of the pores are more than half), incomplete pores and perfect pores corresponding to the 3 cases describing in (c).

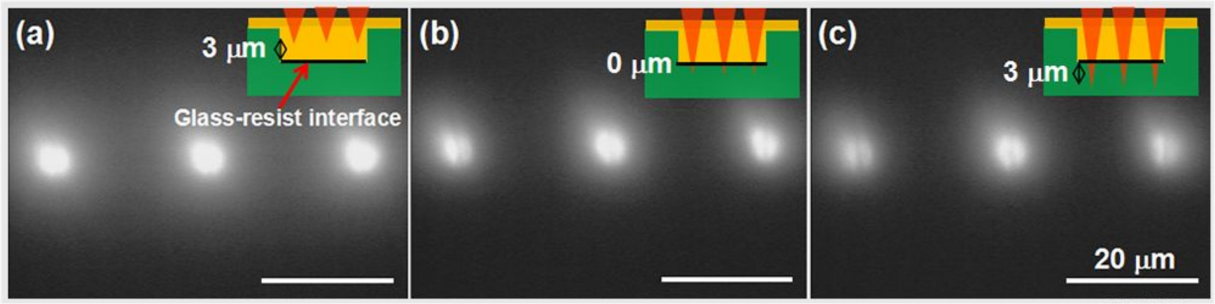

Figure S6. The foci captured by a CCD camera. (a) shows that foci with strong fluorescence locate in the resist. (b) shows that foci with weak fluorescenece locate in the glass-resist interface. (c) shows that foci with weaker fluorescenece locate in the glass. The position of foci in (c) is 3 μm lower than (b). And the position of foci in (a) is 3 μm higher than (b).

(3) The last problem is the uniformity of the pores size. In fact, the filter with inhomogeneous pores (larger pores in the below and smaller pores in the above) can be created by layer-by-layer scanning (Fig. 4a). The foci energy was larger in the above than in the below (Fig. 4c) because of the energy attenuation which was caused by the thick resin (the resin has an imperfect light transmission less than 100%). The larger laser energy will created larger voxels (Fig. 4c) which will create smaller pores or no pores (Fig. 4d). To deal with this problem, we optimized the design of the filter by compensating the pores size reversely (Fig. 4b). Larger pores in the above and smaller pores in the below (Fig. 4b) with appropriate sizes were predesigned, then appropriate laser energy was used. The filters with almost the same pores size can be created by this method which can be seen in the SEM pictures (Fig. 4e).

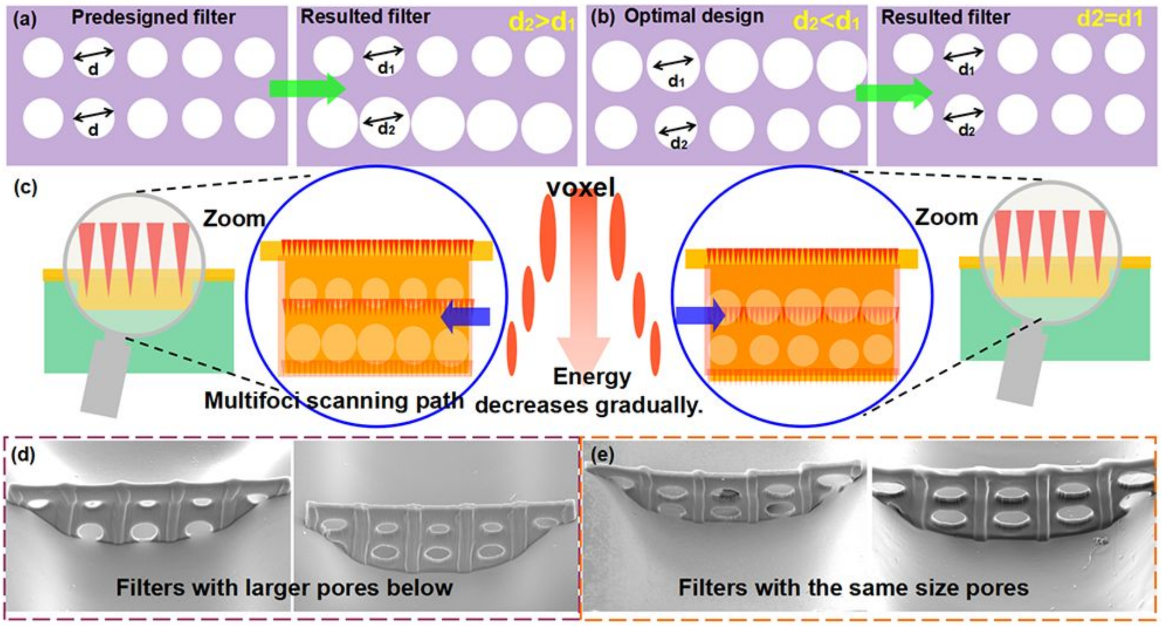

Figure S7. Optimization for uniformity of pores size. (a) shows the first design of the filter. After scanning layer-by-layer, the resulted filter has different size of pores. The pores above are smaller than below. (b) is the optimal design of the filter with pores above larger than below. The resulted filter has a approximate same size pores. (c) explains the reason that the resin decreases the laser energy, and the voxel at the lower position is smaller than that at the upper position. (d) and (e) show SEM images of the resluted filters with different pores produced by the first desigh and uniform pores created by our optimal desigh, respectively.

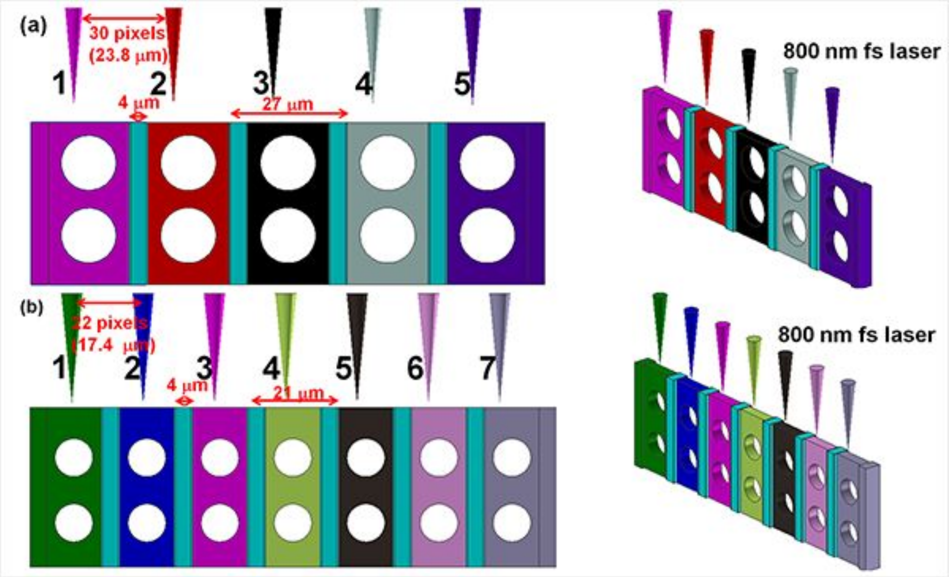

Figure S8. Schematic illustration of the design and fabrication of the filters using fivefoci (a) and sevenfoci (b) were showed. The green parts represented the overlap zone which was scanned twice. The relationship between the distance L of multiple foci and the interval of pixels Δd is expressed as below:

$$L = \frac{\lambda f_1}{d_{pixel} \cdot N_{pixel}} \cdot \Delta d \cdot \frac{f_{objective}}{f_2}$$

where λ is the wavelength (800 nm) of laser source. f1 and f2 are the focal lengths (600 mm and 200 mm) of Lens1 and Lens2 (Fig. S2), respectively. $f_{objective}$ represents the focal length (3.24 mm) of the objective. d_{pixel} is the pixel pitch (8 μm) and N_{pixel} is the number (1080) of pixels. The calculated result shows one pixel in laser pattern corresponds to 0.83 μm in photoresist. So it is very convenient to design a desired multifoci pattern. For 2D and 3D integration, spots interval of 20 pixels was designed (corresponding to 16.6 μm in photoresist) which well agreed with the SEM images (15.9 μm). Figures S4 (a) and (b) show the design of five foci integration of microfilter. Spots interval of 30 pixels were chose which corresponded

23.8 μm of foci interval. The scanned microstructure with a length of 27 μm and two standing columns with a width of 4 μm were designed. From the above design, we can see there is a 3.2 μm length of overlap fabrication zone locating in the columns which will further improve the robustness of the filter. Also, in order to fabricate seven-foci filter, spots interval of 22 pixels was chose, which corresponds 17.4 μm of foci interval. The scanned microstructure with a length of 21 μm and two standing columns (4 μm length) was designed. So, there is a 3.6 μm length of overlap fabrication zone locating in the columns which will also improve the robustness of the filter.

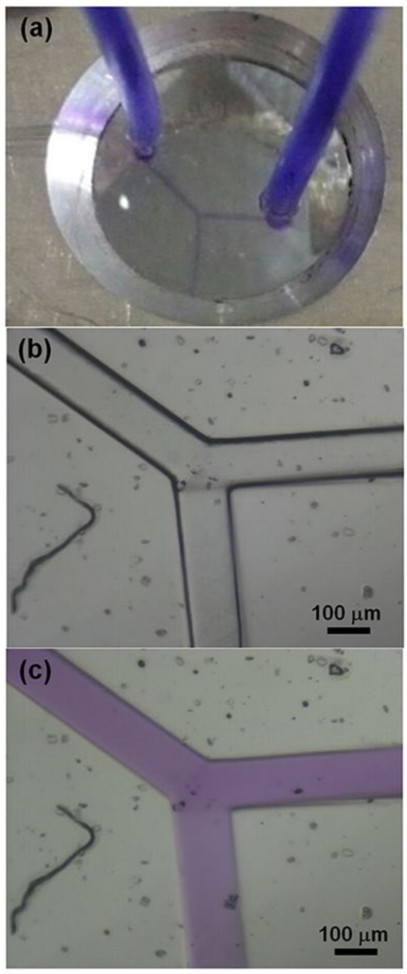

Fig. S9 The alcohol with purple food dye as a media fluid to verify that the microfluidic chip is sealed completely. (a) The glass was covered with a PDMS slab with a inlet and a outlet. When the channel was filled with liquid, we can find that there are no leakage. (b) Naked channel. (c) shows the 20X bright-field microscope images of the ‘Y’ microchannel which is full of the fluid. The above two pictures further prove no leakage of the final microfluidic chip.

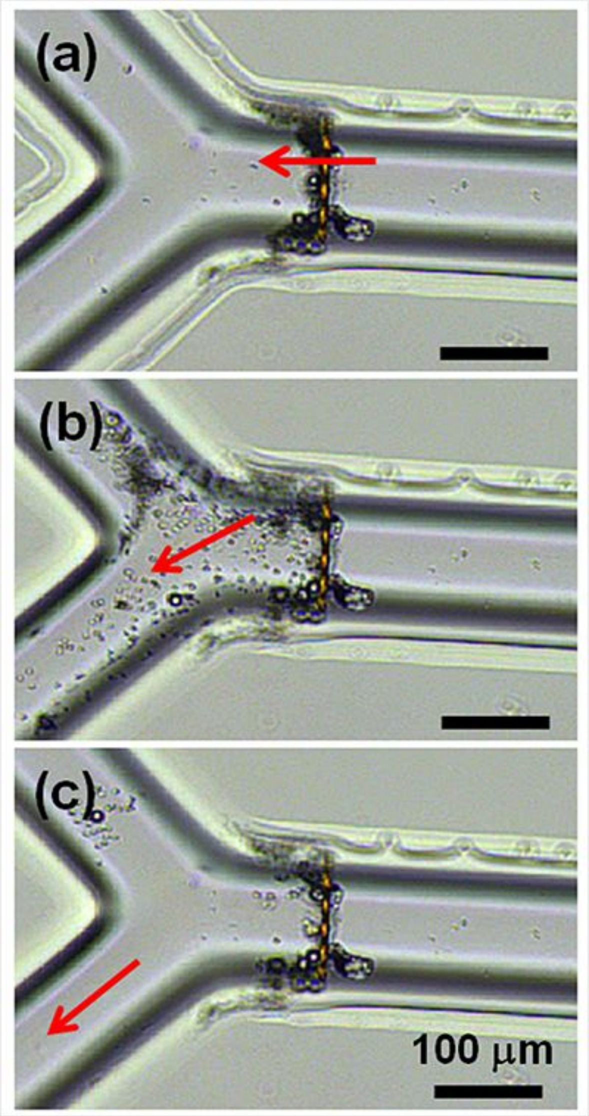

Fig. S10 Chip cleaning. (a), (b) and (c) shows reversely flowing the alcohol to clean the chip. After a long operation time, the filter is blocked by the PS particles which led to the flow rate decreasing, so the particles could not pass through the filter (a). By injecting alcohol solution from the outlet, the particles (adhereing to the filter and the channel walls) will flow again and leave the filter or channel walls away (b). After a while, the filter was cleaned (c). The filter still stood in the channel proving its robust.

Structures / Time(min)	LOC	Cross	Svastika	Layered structures	Hierarchical pillars	Springs	Fivefoci filter	Sevenfoci filter
Single focus	1.53	12.78	23.67	99.27	79.92	41.13	75	63
multifoci	0.17	1.42	2.63	11.03	8.88	4.57	15	9

Table S1. Comparison of the fabrication time of single focus and multifoci. From the table, we can see that multifoci fabrication will greatly reduce the time and enhance the fabrication speed.

Supporting Video 1. The alcohol with purple food dye as a media fluid to verify that the microfluidic chip was sealed completely. The glass microchannel was covered with a PDMS slab. When the channel was filled with liquid, we can find that there are no leakage.

Supporting Video 2. The filtering function (PS particles) of a 12.2 μm-pore microfilter. From the video, the smaller particles (5.2 μm and 2.8 μm PS particles) pass through the filter while the larger ones (13.0 μm PS particle) are blocked with 100%-success rate, presenting an excellent filtering capability.

Supporting Video 3. Chip cleaning by reversely flowing the alcohol into the chip. After a long operation time, the filter was blocked by the PS particles which led to the decreasing flow speed, and the particles could not pass through the filter. Cleaning was performed by reversely injecting alcohol solution with a syringe. After a while, the particles can be cleaned out of the filter and the channel. After cleaning, the filter can be used once again.

Supporting Video 4. Filtering the cancer cells by filter. SUM 159 cancer cells with different sizes flowed to the filter. The larger cells were blocked because they had larger size of nucleis which were much more difficult to be deformed than the cytoplasm. On the contrary, the smaller cells passed through the filter because of the cell deformable ability.
